# Supplementary material for: Chromothripsis during telomere crisis is independent of NHEJ, and consistent with a replicative origin
Source: Genome Res. 2019 May;29(5):737–49. doi: 10.1101/gr.240705.118 (PMC6499312; doi:10.1101/gr.240705.118)
Supplement: Supplemental Material [file supp_gr.240705.118_Supplemental_file_1.zip › contigs/annotated_contigs/DB50/contig.2.DB50_length_730_mean_cov_6.67123287671.docx]

**DB50_length_730_mean_cov_6.67123287671**

```
AAGGCAGCCAGAAAGCAGCGGCC|AGTTACCTACAGAAGAAATCCCACAGTCTAGCAGCAGACATTTCAGCAGAATCCTTACAAGCCAG
                        >chr2:185971195-185971478 + E=2e-155
AAGAGACTTTCAGCATCCTTAAAGAAAAGAAATTCCAATCAAGAATTTCATATCCTGCCAAACTAAGCTTCACAAGTGAAGGAAAAATA

GAATCCTTCTCCAGCAAGCAACTGCTGAGGGAATTCATTTCAACCAGGCCACAATTACAAGAGGTCTTTAAGGGACTGCTAAACATGGA

ATTGAAAAAAATGTCACATGCTACCACAAAAAAAAAAAAA|CACTTAAGCGCATAG|CTCAAGAAAATACTGGCAAATTGAATTCAGCA
                                                        >chr2:185973196-185973605 + E=3
GCACATTAATTCATCATAATCAACTAGACCTAATTCCAGGGATTCAAGGTTGAATCAATATATGCAAAACAATAAAGATGATTGACCAC
e-225
CTAAACAGAATTAAAATAAAAAATCATATGATCATCTCAATAGATATAGAATAAGCTTTCGATAAATCCCAACATCTCTTCATAATAAA

AACCATCAACACACTGGGCATTGAAGGAATATACCTTAAAATCATAAGAGCTATCTATGACAAACCCACAGCGCACATCACACTGAATG

GGCAAAAGGTGGAGCCGTTTCCCTTAAGAAGTGGAACTCTCATCACTCTTATTCAACATAGCACTGGAAGTCCTAGTCAGAACAGCCAG

GCAAGATGAATAAATAAAAGG
```
